# Body Mass Index: A Risk Factor for Retinopathy in Type 2 Diabetic Patients

**DOI:** 10.1155/2013/436329

**Published:** 2013-11-20

**Authors:** Snježana Kaštelan, Martina Tomić, Antonela Gverović Antunica, Spomenka Ljubić, Jasminka Salopek Rabatić, Mirela Karabatić

**Affiliations:** ^1^Department of Ophthalmology, Clinical Hospital Dubrava, Avenija Gojka Šuška 6, 10000 Zagreb, Croatia; ^2^Department of Ophthalmology, University Clinic Vuk Vrhovac, Clinical Hospital Merkur, Zajčeva 19, 10000 Zagreb, Croatia; ^3^Department of Ophthalmology, General Hospital Dubrovnik, Dr. Roka Mišetića 2, 20000 Dubrovnik, Croatia; ^4^Department of Endocrinology and Metabolic Diseases, University Clinic Vuk Vrhovac, Clinical Hospital Merkur, Zajčeva 19, 10000 Zagreb, Croatia; ^5^University of Applied Sciences Velika Gorica, Zagrebačka Cesta 5, 10410 Velika Gorica, Croatia

## Abstract

The aim of the study was to investigate whether body mass index (BMI) independently or in correlation with other risk factors is associated with diabetic retinopathy (DR) progression. The study included 545 patients with type 2 diabetes. According to DR status, they were divided into three groups: group 1 (no retinopathy; *n* = 296), group 2 (mild/moderate nonproliferative DR; *n* = 118), and group 3 (severe/very severe NPDR or proliferative DR; *n* = 131). Patients without DR were younger than those with signs of retinopathy at time of diabetes onset whilst diabetes duration was longer in groups with severe NPDR and PDR. DR progression was correlated with diabetes duration, BMI, HbA_1_c, hypertension, and cholesterol. Statistical analyses showed that the progression of retinopathy increased significantly with higher BMI (gr. 1: 26.50 ± 2.70, gr. 2: 28.11 ± 3.00, gr. 3: 28.69 ± 2.50; *P* < 0.01). We observed a significant deterioration of HbA_1_c and a significant increase in cholesterol and hypertension with an increase in BMI. Correlation between BMI and triglycerides was not significant. Thus, BMI in correlation with HbA_1_c cholesterol and hypertension appears to be associated with the progression of DR in type 2 diabetes and may serve as a predictive factor for the development of this important cause of visual loss in developed countries.

## 1. Introduction 

Overweight (body mass index, BMI ≥ 25 kg/m^2^) and obesity (BMI ≥ 30 kg/m^2^) have become a growing global public health problem with increasing prevalence in many affluent societies as well as in developing countries [1–3]. Currently, 300 million people are considered to be obese and due to this rising trend, it is anticipated that this figure could double by the year of 2025. Addressing the problem of obesity becomes important since being a disease itself it represents a risk for many metabolic and cardiovascular diseases including type 2 diabetes [4]. The number of patients with type 2 diabetes is rapidly increasing in many countries around the world irrespective of its phase of development. It is projected that by 2025 there will be 380 million people with type 2 diabetes and 418 million people with impaired glucose tolerance owing to an increase in obesity, inactivity, life span extension, and better detection of the disease [5]. This global increase of diabetes incidence has a significant impact on the prevalence of diabetic complications among which diabetic retinopathy (DR) takes an important place [6, 7]. DR is a leading cause of acquired blindness in working-age adults and has been estimated to represent 12% of blindness in developed countries [8, 9]. The diagnosis of type 2 diabetes is often preceded by years of undiagnosed hyperglycaemia. Thus, in a number of patients, DR is present at the time of diagnosis: 37% of them already having microaneurisms or more severe retinopathy in one and 18% having retinopathy in both eyes [10, 11]. The prevalence of retinopathy increases with the duration of diabetes and is related to hyperglycemia, hypertension, hyperlipidemia, pregnancy, nephropathy, and anemia [12–14]. 

Since DR has become a main cause of vision loss and blindness worldwide, intense focus on the early prevention of DR and the benefit of controlling modifiable risk factors has become increasingly important. Numerous population-based studies and clinical trials have confirmed that longer duration of diabetes, poor glycemic control, and increased blood pressure (BP) are the key risk factors for the development and progression of DR [12, 13]. However, evidence from new recent trials such as the Action in Diabetes and Vascular Disease (ADVANCE) [15] and the Action to Control Cardiovascular Risk in Diabetes (ACCORD-Eye) [16] has nonetheless shown that risk reduction for DR with better glucose and BP management has been limited. Thus, a better understanding of the role of other modifiable risk factors including obesity in the development and progression of DR becomes even more valuable.

The evidence supporting a relationship between high BMI and increased risk of DR is inconclusive [17–31]. Some studies have demonstrated a relationship between obesity or higher BMI and an increased risk of DR [19–27], whereas others have reported conflicting results [28–31].

Considering that obesity is becoming increasingly prevalent in today's society and since it can be managed by lifestyle intervention, namely, nutrition, exercise, and education studying, its impact on diabetic complications has certain logic and benefits. Thus, the aim of the present study was to investigate whether obesity independently or in association with other established risk factors influences DR development in type 2 diabetic patients. 

## 2. Patients and Methods 

 This cross-sectional study as performed in collaboration with the Ophthalmology Departments of three Croatian Hospitals in accordance with the Declaration of Helsinki and approved by the Ethics Committee of each Hospital. The patients included in the study received both written and oral information concerning the study and signed a written informed consent. 

### 2.1. Patients

A total of 545 patients with type 2 diabetes were included in the study. They were taking either oral hypoglycemic agent (OHA) or insulin therapy. Type 2 diabetes was defined according to the American Diabetes Association classification [32]. Patients with malignancies, immunologic, and infectious inflammatory diseases as well as patients receiving corticosteroids or cytostatics, pregnant women, and patients with other eye diseases (mature cataract, uveitis, age-related macular degeneration) were excluded from the study. 

### 2.2. Methods

 This study included diabetic patients who attended their regular medical and ophthalmological checkups for six months period. Patients who met all inclusion criteria were invited to participate in the study and signed the consent form. Blood samples for laboratory analyses were collected between 08:00 and 10:00 am after a 12 hour overnight fast and complete clinical and ophthalmic examinations were performed. 

#### 2.2.1. Blood Samples

Glycated hemoglobin value (HbA_1_c), total cholesterol, and triglycerides were measured. HbA_1_c was determined by an automated immunoturbidimetric assay (reference values 3.5–5.7%) [33]. Total cholesterol and triglycerides were measured by the enzymatic colorimetric tests (reference values: total cholesterol < 5.00 mmol/L; triglycerides < 1.70 mmol/L) [34, 35]. 

#### 2.2.2. Anthropometric Parameters

BMI as a common index of obesity was calculated by dividing weight and height squared (kg/m^2^). Weight was measured using a balance-beam scale and height was measured using a wall-mounted stadiometer with patients in their underwear and without shoes. Recommended value of BMI among men was considered <23 and among women <22 kg/m^2^ with a normal range being between 18.5 and 24.9 kg/m^2^ [36].

#### 2.2.3. Clinical Parameters

Blood pressure was measured with an ambulatory sphygmomanometric device after a 5 min rest and the mean of three measurements was used. Hypertension was defined as blood pressure > 130/80 mmHg or the use of antihypertensive treatment.

#### 2.2.4. Ophthalmologic Examination

Complete eye examination included best corrected visual acuity (BCVA), Goldmann applanation tonometry, slit lamp biomicroscopy of the anterior eye segment, binocular indirect slit lamp fundoscopy, and fundus photography after mydriasis with topically administrated 1% tropicamide and 5% phenylephrine eye drops. Color fundus photographs of two fields', namely, macular field and disc/nasal field of both eyes, were taken with a suitable 45° fundus camera (VISUCAM, Zeiss) according to the Europe and Diabetes Study (EURODIAB) retinal photography methodology [37]. Macular field: where the exact centre of the optic disc is laid at the nasal end of the horizontal meridian of the field view. Disc/nasal field: where the optic disc is positioned one disc-diameter in from the temporal edge of the field on the horizontal meridian. The EURODIAB classification scheme was applied since it uses two-field 45° fundus photography and standard photographs to grade retinal lesions [38]. According to the DR status, patients were divided into three groups: group 1 (no retinopathy; *n* = 296), group 2 (mild/moderate nonproliferative DR (NPDR); *n* = 118), and group 3 (severe/very severe NPDR or proliferative diabetic retinopathy (PDR); *n* = 131). The severity of retinopathy was determined according to the status of the seriously affected eye using fundus photographs.

### 2.3. Statistical Analyses

For all, analyzed variables descriptive statistics (*n*, mean ± standard deviation) were done. Comparison between groups was performed using ANOVA analysis of variances [38]. Nominal scaled data was tested with the chi-square-test (*χ* ^2^). The relationship between DR, obesity (defined by BMI), and risk factors was analyzed using Pearson's correlation test. To test the hypothesis of independence features, the chi-square-test (*χ* ^2^) was conducted. Data were analyzed with IBM SPSS software version 12.0 [39]. In all the analyses, *P* value of less than 0.01 and 0.05 were considered statistically significant.

## 3. Results

This study included 545 patients with type 2 diabetes (280 male (51%), 265 female (49%)) with a mean age of 68.28 ± 8.99 years. The mean age at onset was 55.26 ± 9.86 years and the mean duration of diabetes was 12.97 ± 6.51 years. According to the diabetic retinopathy status, they were divided into three groups: group 1 (no retinopathy; *n* = 296), group 2 (mild/moderate NPDR; *n* = 118), and group 3 (severe/very severe NPDR or proliferative diabetic retinopathy (PDR), *n* = 131). Incidence of any form of retinopathy in the examined patients (*n* = 545) was 46% of which 19% had severe to very severe NPDR and 5% had PDR.

Table 1 presents descriptive statistics of basic characteristics of type 2 diabetic patients divided into three groups according to DR status. There was no difference in gender between the investigated groups. Group 2 was found to be significantly older than groups 1 and 3 (70.87 ± 7.8 years versus 67.68 ± 9.6 and 67.28 ± 8.1 years; *P* < 0.01), whilst group 3 was younger than groups 1 and 2 when diabetes was diagnosed (51.44 ± 9.2 years versus 56.94 ± 9.8 and 55.29 ± 9.6 years; *P* < 0.01). Duration of diabetes was significantly longer in groups 2 and 3 than in group 1 (15.79 ± 7.5 and 15.73 ± 6.4 years versus 10.62 ± 5.1 years; *P* < 0.01). 

Body mass index was significantly higher in groups 2 and 3 than in group 1 (28.11 ± 3.0 and 28.69 ± 2.5 kg/m^2^ versus 26.50 ± 2.7 kg/m^2^; *P* < 0.01) (Table 2). 

In order to analyze other factors that have an influence on the development of DR, we classified metabolic and clinical parameters in the investigated groups according to DR status (Table 3). We observed a significant deterioration of HbA_1_c (*P* < 0.01) and a significant increase in total cholesterol (*P* < 0.01), triglycerides (*P* < 0.05) as well as systolic (*P* < 0.01) and diastolic blood pressure (*P* < 0.01) with the progression of retinopathy.

DR was marginally negatively correlated with the onset of diabetes (*P* = −0.120), whilst a significantly positive correlation with diabetes duration (*P* = 0.377), BMI (*P* = 0.338), HbA_1_c (*P* = 0.583), total cholesterol (*P* = 0.281), and systolic (*P* = 0.446) and diastolic blood pressure (*P* = 0.430) was observed. The correlation between DR and triglycerides was marginally positive (*P* = 0.115). These data are shown in Table 4.

BMI was significantly positively correlated with HbA_1_c (*P* = 0.674) and systolic (*P* = 0.509) and diastolic blood pressure (*P* = 0.435), whereas marginally positively correlated with the onset of diabetes (*P* = 0.209) and total cholesterol (*P* = 0.110). There was no correlation between BMI and triglycerides (*P* = 0.077). These data are shown in Table 5.

Table 6 presents the significant and independent association of BMI and the prevalence of DR in type 2 diabetic patients (*P* < 0.01) evaluated by *χ* ^2^ hypothesis testing.

## 4. Discussion

Type 2 diabetes represents one of the most detrimental diseases and significant public health problems due its high incidence and prevalence as well as high risk of diabetic macro- and microvascular complications. DR, a sight threatening microvascular complication of diabetes, is the leading cause of blindness and visual dysfunction in the working age population in developed countries [7, 8]. According to a study conducted to estimate the global prevalence of DR, 35% of people with diabetes had some form of DR, 7% had PDR, 7% had DME whilst 10% had vision threatening DR, defined as the presence of PDR and/or DME [40]. Prevalence of DR in individuals with long-standing diabetes (20 years or more) exceeded 50% [41]. In our investigated group, the mean duration of diabetes was 12.97 ± 6.51 years with prevalence of any form of retinopathy being 46% whilst 19% of the patients had severe to very severe NPDR and 5% of them had PDR. 

Many previous epidemiological and clinical studies have shown that diabetes duration and prolonged poor glycemic control are the main predictors of the prevalence and progression of retinopathy in patients with type 2 diabetes [21, 42, 43]. The results of our study are consistent with these findings. Significant differences in the onset and the duration of diabetes between our investigated groups were observed with advanced stages of DR in those patients with a longer duration of diabetes. Furthermore, the obtained results indicated the existence of statistically significant differences for HbA_1_c, total cholesterol and triglycerides between the investigated groups. In addition to quality of metabolic control, hypertension is another important risk factor for DR development which is documented in many epidemiological studies and clinical trials. The United Kingdom Prospective Diabetes Study (UKPDS) and Appropriate Blood Pressure Control in Diabetes (ABCD Study) have observed that strict blood pressure control can prevent and/or limit the development and progression of DR and visual dysfunction [44, 45]. Our investigation indicated a significant difference in the level of systolic and diastolic blood pressure between the groups according to their DR status. It is worth noting that in our patients without retinopathy, the average systolic blood pressure was 138.24 ± 11.05 mmHg and the average diastolic blood pressure was 83.94 ± 5.71 mmHg with these values being very close to those recommended by the American and European Societies of Cardiology [46, 47]. According to our results, DR was significantly positively correlated with diabetes duration (*P* = 0.377), BMI (*P* = 0.338), total cholesterol (*P* = 0.281), HbA_1_c (*P* = 0.583), and systolic (*P* = 0.446) and diastolic blood pressure (*P* = 0.430) whilst the correlation with triglycerides was marginally positive (*P* = 0.115). 

Good glycaemic control and strict treatment of hypertension significantly reduces the risk of microvascular complications [11, 48–50] and therefore remains the best available treatment strategy to inhibit or prevent the development of DR. However, tight control of these risk factors certainly may reduce but not completely eliminate the risk of retinopathy, it opens up a continuing need for the development of new intervention strategies. These potential interventions are limited by a deficiency of knowledge about the presumed risk factors for retinopathy, their precise effects, and interrelationship. Therefore, in addition to the well known risk factors, increasing attention is assigned to obesity specifically due to its frequency and interrelationship with type 2 diabetes. Obesity intensifies the risk of type 2 diabetes, its macrovascular complications, and reduces life expectancy in all age groups [4, 51]. An increase in BMI also correlated significantly with the deterioration of HbA_1_c, a decrease in HDL-cholesterol, an increase in triglycerides as well as a higher prevalence of hypertension [24, 52]. The results of our study also demonstrated a positive correlation between BMI and investigated risk factors namely HbA_1_c (*P* = 0.674), total cholesterol (*P* = 0.110), and systolic (*P* = 0.509) and diastolic blood pressure (*P* = 0.435) with the strongest association being with HbA_1_c.

The relationship between BMI and DR has been examined in a number of epidemiologic studies yielding inconsistent results. Most studies have reported a significant association between high BMI and obesity with DR [19–27]. Conversely, others have reported an association between low BMI and DR [29–31] suggesting a possible protective role for higher BMI in the development of DR. This lack of consensus may be partly explained by methodological differences, differences in study participants, lack of comprehensive anthropometric measurements, inadequate clinical sample size, and particularly racial or ethnic differences since a negative correlation of BMI and DR was found in Asian populations [29, 30]. Our study findings concur with those studies that have reported an increased risk of DR in patients with high BMI where we also found a significant difference between investigated groups with the higher value being in those with advanced stages of DR. 

Although the underlying pathophysiological mechanisms supporting the association between higher BMI and DR are yet to be defined, several biological theories have been proposed including the potential involvement of platelet function, blood viscosity, aldose reductase activity, and vasoproliferative parameters such as vascular endothelial growth factor (VEGF). Furthermore, both metabolic syndrome and increased oxidative stress due to their association with obesity and DR have also been suggested as possible pathophysiological mechanisms [17, 53, 54]. Recently, increasing interest has been directed to the role of vasoproliferative factors particularly the VEGF in the pathogenesis of DR. The concentration of VEGF has been found to be higher in the vitreous of eyes with PDR [55]. Likewise in the serum of obese individuals', elevated angiogenic factors including VEGF have been observed partially owing to the presence of oxidative stress [56] providing additional confirmation of the possible link between obesity and PDR.

Furthermore, obesity increases the prevalence of several risk factors involved in DR onset and development including inflammatory markers. New data suggests that obesity is associated with both increased local adipose and more generalized systemic inflammation. Adipose tissue is considered to be an active endocrine and paracrine proinflammatory organ that releases a large number of cytokines and bioactive mediators, namely, leptin, adiponectin, interleukin-6 (IL-6), tumour necrosis factor-*α* (TNF-*α*) that influence not only body weight homeostasis but also lipid levels, coagulation, atherosclerosis and diabetes occurrence, insulin resistance, inflammation, oxidative stress, and DR development. Moreover, endothelial dysfunction as an early marker of DR is also present in obesity and is characterised by increased levels of intracellular adhesion molecule-1 (ICAM-1) [57–60].

Ultimately, obesity and DR may also be connected owing to increased oxidative stress as a result of its association with hyperleptinemia [18, 61]. Plasma leptin levels are seen to be elevated in obesity and correlate positively with both visceral and subcutaneous fat areas [62]. High plasma leptin level have been found to relate to both hypertensive and diabetic retinopathy [61]. Pertinent to DR, recent findings show that leptin promotes vascular endothelial cell proliferation and angiogenesis in vitro and neovascularization in vivo [27, 63]. Conversely, adiponectin levels correlate negatively with visceral and subcutaneous fat areas [62], whilst low adiponectin levels are associated with obesity and insulin resistance [27]. It is observed that adiponectin values correlate negatively with BMI [64], waist-to-hip ratio and plasma triglycerides are yet to have a positive correlation to HDL cholesterol [27, 64].

Epidemiological data from various studies have identified hyperlipidemia and hypertension which are connected with obesity as risk factors for DR [17, 24, 65–67]. In fact, metabolic syndrome encompassing these conditions has also been shown to be associated with retinopathy [68]. Summarizing these facts, obesity and associated insulin resistance may increase the risk of DR development via several established mechanisms, namely, dyslipidemia hypertension and glucose dysmetabolism as well as some new mechanisms, whose roles still need to be clarified such as leptin, adiponectin, IL-6, TNF-*α* and ICAM-1. Mutually, they may lead to an increase in oxidative stress, endothelial dysfunction, and finally DR development [60, 69]. Furthermore, physical activity and weight loss as lifestyle factors provide some additional evidence to support the relationship between high BMI and DR, whereby weight loss has been seen to delay the onset of diabetic complications including DR [70].

Our main study findings show that BMI is associated with the presence and severity of DR and thus additionally confirms the results of the majority of previously conducted studies opening up implications for further research and intervention. The striking associations between BMI and DR indicate that there is a unifying feature of these factors which account for their relationship with retinopathy. Taking this into account while applying the interventions that reduce BMI may consequently also reduce the risk of DR. The impact of weight loss on regression of retinopathy particularly in obese individuals for the time being has been inadequately investigated [17]. Lifestyle changes specifically weight loss have been advocated as a key factor in helping prevent diabetes and to delay diabetic complications including retinopathy in susceptible patients [17, 71]. Regardless of insufficient data, it is generally accepted that weight reduction should certainly be advised in obese diabetic patients in order to reduce the risk of cardiovascular disease [69, 72, 73] and possibly DR [73].

In conclusion, DR is a complex disease with several proven and some insufficiently verified proposed risk factors including inflammation. We have shown that BMI in correlation with poor glycaemic control, hypertension, and dyslipidemia appears to be associated with the progression of DR in type 2 diabetes. Accordingly, it may be a relevant factor in a cascade involving the occurrence and development of DR. Our findings confirm the results of most of the previous investigations and illustrate the value of obesity assessment as an important modifiable risk factor which may consequently have potential clinical implications in the management of DR. Since weight gain is changeable and may be managed by lifestyle intervention, additional studies are required to investigate and fully elucidate the pathogenic role of obesity and weight loss in retinal diabetic complications. Current knowledge and assumptions pertaining to the pathophysiological mechanism of BMI in DR development open up the need for further research to clarify the role of inflammation and body weight and their interaction on the pathogenesis of DR.

## Figures and Tables

**Table 1:** Basic characteristics of type 2 diabetic patients (*n* = 545) divided into three groups according to diabetic retinopathy status.

	Group 1 (*n* = 296)	Group 2 (*n* = 118)	Group 3 (*n* = 131)	*P*
Sex (m/f)*	51.35/48.65	45.76/54.24	56.49/43.51	
Age (years)**	67.68 ± 9.6	70.87 ± 7.8	67.28 ± 8.1	<0.01
Onset of diabetes (years)*	56.94 ± 9.8	55.29 ± 9.6	51.44 ± 9.2	<0.01
Diabetes duration (years)**	10.62 ± 5.1	15.79 ± 7.5	15.73 ± 6.4	<0.01

*(%); **mean ± SD.

Group 1: no retinopathy, Group 2: mild/moderate nonproliferative diabetic retinopathy, and Group 3: severe/very severe nonproliferative diabetic retinopathy or proliferative diabetic retinopathy.

**Table 2:** Body mass index (BMI) of type 2 diabetic patients (*n* = 545) divided into three groups according to diabetic retinopathy status.

	Group 1 (*n* = 296)	Group 2 (*n* = 118)	Group 3 (*n* = 131)	*P*
BMI (kg/m^2^)*	26.50 ± 2.7	28.11 ± 3.0	28.69 ± 2.5	<0.01

*Mean ± SD.

Group 1: no retinopathy, Group 2: mild/moderate nonproliferative diabetic retinopathy, and Group 3: severe/very severe nonproliferative diabetic retinopathy or proliferative diabetic retinopathy.

**Table 3:** Metabolic and clinical parameters of type 2 diabetic patients (*n* = 545) divided into three groups according to diabetic retinopathy status.

	Group 1 (*n* = 296)	Group 2 (*n* = 118)	Group 3 (*n* = 131)	*P*
HbA_1_c (%)*	7.14 ± 0.9	7.96 ± 1.1	8.92 ± 1.4	<0.01
Total cholesterol (mmol/L)*	4.60 ± 0.9	4.77 ± 1.1	5.24 ± 1.1	<0.01
Triglycerides (mmol/L)*	1.44 ± 0.7	1.50 ± 0.5	1.59 ± 0.53	<0.05
Systolic blood pressure (mmHg)*	138.24 ± 11.5	145.93 ± 11.7	151.95 ± 13.14	<0.01
Diastolic blood pressure (mmHg)*	83.94 ± 5.71	86.53 ± 6.2	91.30 ± 8.05	<0.01

*Mean ± SD.

Group 1: no retinopathy, Group 2: mild/moderate nonproliferative diabetic retinopathy, and Group 3: severe/very severe nonproliferative diabetic retinopathy or proliferative diabetic retinopathy.

**Table 4:** Correlation between diabetic retinopathy and diabetes duration, body mass index, metabolic, and clinical parameters in type 2 diabetic patients (*n* = 545).

	Diabetic retinopathy
	Correlation test*
Onset of diabetes	−0.120**
Diabetes duration	0.377**
Body mass index (BMI)	0.338**
HbA_1_c	0.583**
Total cholesterol	0.281**
Triglycerides	0.115**
Systolic blood pressure	0.446**
Diastolic blood pressure	0.430**

**Correlation is significant at the 0.01 level (2-tailed).

**Table 5:** Correlation between body mass index and metabolic and clinical parameters in type 2 diabetic patients (*n* = 545).

	Body mass index (BMI)
	Correlation test*
Onset of diabetes	0.209*
HbA_1_c	0.674**
Total cholesterol	0.110*
Triglycerides	0.077
Systolic blood pressure	0.509**
Diastolic blood pressure	0.435**

**Correlation is significant at the 0.01 level (2-tailed).

*Correlation is significant at the 0.05 level (2-tailed).

**Table 6:** Diabetic retinopathy in type 2 diabetic patients (*n* = 545) divided into three groups according to their body mass index (BMI).

Retinopathy	BMI (kg/m^2^) ≤25.0	BMI (kg/m^2^) 25.1–29.9	BMI (kg/m^2^) ≥30.0	*∑*
No retinopathy	80	190	26	296
Retinopathy	22	158	69	249

*∑*	102	348	95	545

BMI: body mass index.
